# Macrophage Rewiring by Nutrient Associated PI3K Dependent Pathways

**DOI:** 10.3389/fimmu.2019.02002

**Published:** 2019-08-21

**Authors:** Omar Sharif, Julia Stefanie Brunner, Andrea Vogel, Gernot Schabbauer

**Affiliations:** ^1^Centre for Physiology and Pharmacology, Institute for Vascular Biology, Medical University Vienna, Vienna, Austria; ^2^Christian Doppler Laboratory for Arginine Metabolism in Rheumatoid Arthritis and Multiple Sclerosis, Vienna, Austria

**Keywords:** macrophage, PI3K, nutrient sensing, adipose tissue macrophages, metainflammation, insulin

## Abstract

Class 1 Phosphoinositide-3-Kinases (PI3Ks) have been widely studied and mediate essential roles in cellular proliferation, chemotaxis, insulin sensitivity, and immunity. Here, we provide a comprehensive overview of how macrophage expressed PI3Ks and their downstream pathways orchestrate responses to metabolic stimuli and nutrients, polarizing macrophages, shaping their cellular identity and function. Particular emphasis will be given to adipose tissue macrophages, crucial players of insulin resistance and chronic metabolically triggered inflammation during obesity. An understanding of PI3K dependent wiring of macrophage responses is important as this is involved in various diseases ranging from obesity, type 2 diabetes to chronic inflammatory disease.

## Introduction

The PI3K family is a central metabolic regulator, responsible for phosphorylating inositol lipids at the 3′ position of the inositol ring. PI3K generated phosphatidylinositol-3,4,5-trisphosphate (PtdIns(3,4,5)P3) triggers the recruitment and activation of several signaling proteins to the plasma membrane, thereby relaying various extracellular stimuli including Toll-like receptor (TLR) ligands, insulin and G-protein coupled receptor ligands (1, 2). Although there are three classes of PI3K enzymes (3), this mini-review will focus on class I PI3Ks and their function in macrophages in response to metabolic stimuli that are upregulated during obesity, including insulin, glucose, cholesterol and free fatty acids (FFAs). Indeed, macrophages that reside in adipose tissue (ATMs) are exposed to increased levels of these stimuli in the obese state and are significant players in metabolically triggered inflammation (herein referred to as meta-inflammation), which is crucial in the pathogenesis of type 2 diabetes (T2D) and atherosclerosis (4–7). Here, we present an overview of how the aforementioned stimuli regulate macrophage function and propose that PI3Ks are central integrators of these environmental cues.

## The PI3K Pathway and its Effects on Macrophage Polarization

In mammals, class I PI3Ks are subdivided into class IA and class IB. Class IA consists of three catalytic (p110α/β/δ) and five regulatory subunits (p85α/β, p55α/p50α, and p55γ), in part generated through splicing or alternative transcription (p55α/p50α), associated to mainly receptor tyrosine kinases. Class IB only features one catalytic (p110γ) and two regulatory subunits (p84/p101) associated to G-protein-coupled receptors. The catalytic subunit of PI3K heterodimerizes with a regulatory subunit that dictates localization and activity of the complex leading to recruitment of signaling molecules that bind PtdIns(3,4,5)P3 through their pleckstrin-homology (PH) domains including protein kinase B (PKB, also known as AKT), phosphoinositide-dependent kinase 1 (PDK-1), protein kinase C (PKC) and Bruton's tyrosine kinase (BTK). PI3K activation further blocks degradation and increases synthesis of proteins via mTOR signaling. AKT mediates effects involved in glucose transport, glycogen synthesis, and protein synthesis. Some of these metabolic effects are achieved through AKT mediated phosphorylation of Forkhead (FOXO) transcription factors (8–10). Given the crucial role of the PI3K pathway in cellular biology, mechanisms exist to limit its activation. PtdIns(3,4,5)P3 turnover is terminated by lipid phosphatases, such as phosphatase and tensin homolog (PTEN), a prominent tumor suppressor (11).

In macrophages, the PI3K pathway regulates the response to different metabolic and inflammatory signals and modulates macrophage polarization. Briefly, based on their microenvironment and the consequent functional programs elicited, macrophage phenotypes are defined as M1 and M2. Classically activated M1 macrophages adopt a pro-inflammatory phenotype in response to interferon gamma (IFN-γ) and lipopolysaccharide (LPS) and are critical for host defense against pathogens. Alternatively activated M2 macrophages play important roles in wound healing and resolving inflammation. M2 macrophages can further be subdivided into M2a (activated by interleukin (IL)-4 and IL-13), M2b (activated by immune complexes and TLR ligands), and M2c (activated by IL-10 and glucocorticoids). Importantly, these activation states are likely dynamic and influenced by the changing local milieu, therefore macrophages may not form clear cut activation subsets *in vivo* (12). Indeed, as discussed later, recent evidence indicates that ATMs adopt a unique metabolically activated state in response to their microenvironment.

Numerous studies have implicated PI3Ks in limiting pro-inflammatory responses in TLR stimulated macrophages, especially upon LPS mediated TLR4 activation. The mechanisms are diverse ranging from indirect effects such as suppression of TLR4 induced signaling cascades (e.g., MAP kinase signaling) to direct mechanisms, including AKT mediated modulation of FOXO transcription factors or the promotion of M2 responses. Indeed, LPS driven ERK, p38, and JNK pathways in monocytes and macrophages are enhanced upon pharmacological blockage of PI3K activity (13). Bone marrow macrophages (BMMs) deficient in p110γ or p85α exhibit augmented IL-6, IL-12, and TNF levels following LPS challenge, providing genetic evidence that PI3Ks attenuate LPS induced inflammation (14, 15). Further, PTEN deficient macrophages, which exhibit sustained PI3K activity, display decreased LPS driven pro-inflammatory cytokine expression and are skewed toward an M2 phenotype compared to controls (15, 16). In addition, downstream AKT signaling is required for the dampening effects of PI3Ks on TLR4 signaling and might involve phosphorylation and thereby termination of FOXO transcription factor activity. This is particularly important as FOXO1, which when active potentiates TLR4 expression (14, 17). Of note, three distinct isoforms of AKT exist: AKT1, 2 and 3 and studies utilizing AKT isoform-specific deficient mice suggest unique roles for the isoforms in mediating pro and anti-inflammatory signaling (18, 19). LPS stimulated *Akt1*^−/−^ macrophages express augmented levels of iNOS (inducible nitric oxide synthase), NO (nitric oxide), TNFα, and IL-6, whereas LPS treated *Akt2*^−/−^ macrophages produce low levels of these pro-inflammatory mediators suggesting deletion of *Akt1* promotes M1 while deletion of *Akt2* results in M2 responses (20). In line, *Akt2*^−/−^ macrophages express increased levels of the M2 markers arginase 1 (Arg-1), FIZZ1, and exhibit more IL-10 upon LPS treatment compared to controls, while AKT1 deficiency results in enhanced bacterial clearance *in vivo* (20, 21). Interestingly, similar to *Akt2*^−/−^ macrophages the M2 phenotype of *Pten*^−/−^ macrophages is associated with elevated Arg-1 levels that are mediated by binding of the transcription factor CEBP-β to the Arg-1 promoter, suggesting sustained PI3K activity impinges particularly upon AKT1 in the context of macrophage polarization (16, 20). However, whether specific AKT isoforms are regulated by specific PI3K classes remains unknown.

## Adipose Tissue Macrophages

**Figure 1:**
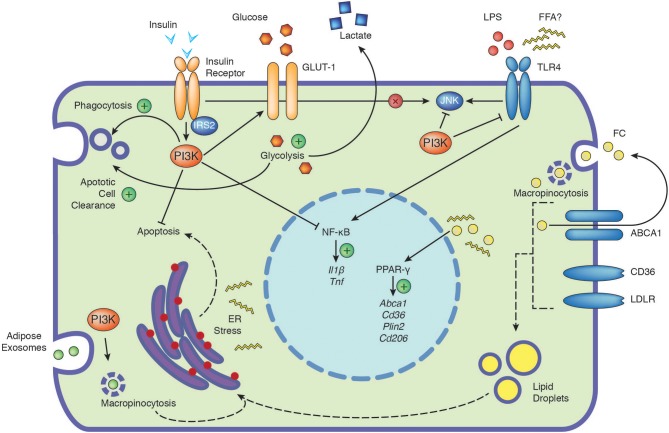
The PI3K signaling cascade integrates signals from extracellular nutrients and influences cellular function. Depicted are the positive (plus) and negative influences of PI3K on the cellular responses to insulin, glucose, TLR4, free cholesterol and adipose exosomes. Insulin signaling in macrophages has no direct impact on JNK activation (cross). Dashed arrows indicate potential connections, see text for further details. ABCA1, ATP-binding cassette 1; CD36, cluster of differentiation 36; CD206, cluster of differentiation 206; ER, endoplasmic reticulum; FC, free cholesterol; FFA, free fatty acids; GLUT-1, glucose transporter 1; IL1β, interleukin 1β; IRS2, insulin receptor substrate 2; JNK, c-Jun N-terminal kinase; LDLR, low-density lipoprotein receptor; LPS, lipopolysaccharide; NF-κB, nuclear factor kappa-light-chain-enhancer of activated B cells; PI3K, phosphoinositide 3-kinase; Plin2, perilipin-2; PPAR-γ, peroxisome proliferator-activated receptors gamma; TLR4, toll-like receptor 4; TNF, tumor necrosis factor.

Although murine ATMs are a heterogeneous population of cells, ATMs in the lean state can generally be described as F4/80^+^CD11b^+^CD206^+^ cells. Physiological adipose tissue growth is associated with minimal inflammation, while during pathological fat expansion, characteristic of obesity, limited angiogenesis of adipose tissue is associated with prevalent adipocyte hypertrophy, fibrosis and death (22). Here, ATM numbers dramatically increase due to local proliferation and recruitment of monocytes into adipose tissue that occurs partly through a monocyte chemoattractant protein 1/C-C chemokine receptor type 2 (MCP-1/CCR2) dependent axis and is influenced by adipose tissue lipolysis (23–26). Indeed, recruited ATMs express CCR2, but also CD11c, CD64, and CD9 (27). CD11c^+^ ATMs overexpress pro-inflammatory genes and ablation of CD11c^+^ cells in adipose tissue of obese mice leads to reduced inflammation and improved insulin sensitivity (28). Nonetheless, while in obesity, recruited ATMs overexpress several classic inflammatory (M1) markers e.g., *Il6* and *Nos2* (29), their phenotype is highly plastic and dependent on the microenvironment. Here, saturated FFAs (e.g., palmitate) or cholesterol, insulin and glucose that are prevalent in obese adipose tissue induce a state of metabolic activation (MMe) in ATMs, distinct from classic M1 activation. MMe activation is associated with elevated cell surface expression of lipid metabolism associated proteins including ATP binding cassette transporter (ABCA1), cluster of differentiation 36 (CD36), and perilipin 2 (PLIN2). This is related to augmented peroxisome proliferator activated receptor gamma (PPAR-γ) binding to the promoters of these genes. Further, autophagy and particularly sequestome-1 (p62) are important as opposed to controls attenuated levels of these lipid mediators occur in p62 null MMe macrophages (30). MMe activation correlates with lysosomal biogenesis as more active biogenesis occurs in newly recruited CD11c^+^ ATMs (27, 31). Recent work has corroborated that ATMs represent a heterogeneous population of cells and that irrespective of obesity, there are populations of lipid laden ATMs associated with the vasculature of adipose tissue exhibiting high endocytic capacity. This suggests active ATM reprograming in response to diverse macromolecules and nutrients present in the bloodstream (32). Together, ATMs respond to their environment by upregulating lipid/lysosomal programs, which is likely heighted during obesity, allowing them to fulfill their main function of clearing up dying adipocytes, buffering lipids, preventing ectopic lipid spill over, and ensuing insulin resistance (25, 26, 33, 34). But how does PI3K activity within macrophages, reconcile with the environmental cues that dictate ATM function and metabolic health? Although cytokines or adipokines secreted by adipose tissue can influence systemic inflammation as well as local macrophage responses (31), here we will focus exclusively on metabolic stimuli relevant to obesity and T2D and their effects on myeloid cells, particularly macrophages (Figure 1). We propose that during obesity, the metabolic milieu encountered by macrophages modulates PI3K signaling driving changes in macrophage function.

## Insulin Stimulated PI3Ks Promote Cell Survival and Attenuates Lipid Loading in Myeloid Cells

Insulin represents an essential hormone for the maintenance of whole-body glucose disposal, regulating carbohydrate, protein and lipid metabolism in insulin-sensitive organs such as adipose tissue, muscle and liver (10). Upon insulin binding, the insulin receptor (IR) self-phosphorylates and activates insulin receptor substrates (IRS) which mediate downstream effects through engaging central signaling pathways including the PI3K/AKT, mTOR, and MAPK pathways (9). In this complex network, PI3Ks are a critical signaling node, mediating many of the metabolic and mitogenic effects of insulin. Importantly, the exact function of insulin signaling in immune cells remains largely unknown, although recently it was shown that T cell specific insulin signaling promotes a specific metabolic program, inducing nutrient uptake to support optimal T cell effector functions (35). Tissue resident macrophages, including ATMs, liver and peritoneal macrophages (pMOs) express the *INSR* gene with pMOs exhibiting highest expression. *INSR* upregulation in pMOs is linked with obesity and M1 macrophages exhibit more expression compared to unstimulated (M0) or M2 macrophages (36). Further, macrophages mainly express IRS-2 but not IRS-1 (37, 38). Although insulin stimulation of macrophages engages the PI3K/AKT signaling cascade (39, 40), it does not activate some important other nodes of the insulin signaling network such as the c-Jun N-terminal kinase (JNK) and p38 pathways (36). Macrophage glucose transport is facilitated mainly via glucose transporter 1 (GLUT1), which is rapidly induced by insulin, an effect that has been described to be more prominent in M1 vs. M0 or M2 macrophages, suggesting possible anti-inflammatory actions of insulin (Figure 1) (36, 41). In line, insulin promotes IL-10 expression dose dependently in pMOs and RAW264.7 macrophages and insulin priming attenuates TLR4 expression, LPS induced nuclear factor kappa B (NF-κB), p38 MAPK activation, and IL-1β production (42). Further, treatment of obese individuals with insulin reverses the pro-inflammatory phenotype of macrophages, eliciting anti-inflammatory effects (43). Concordant with a potential role in resolving macrophage mediated inflammation, insulin-stimulated macrophages exhibit increased expression of phagocytosis associated NAPDH oxidase activity and decreased apoptosis (44, 45). Nonetheless, insulin and PI3K signaling are unlikely to solely promote anti-inflammatory effects. Insulin is reported to increase TNF-α release in human monocytes (46). LPS-stimulation of IR deficient macrophages failed to induce IL-6 and IL-1β expression suggesting insulin signaling might be required for inflammation (47).

In obesity, surprisingly, mice deficient for the IR specifically in myeloid cells exhibit a protective phenotype associated with decreased ATM accumulation and improved insulin sensitivity (48). A recent report has reproduced these findings, additionally suggesting that there are less pro-inflammatory (F4/80^+^CD11c^+^CD206^−^) and more anti-inflammatory (F4/80^+^CD11c^−^CD206^+^) ATMs present in obese mice lacking the IR in myeloid cells, proposing myeloid cell specific IR signaling modulates ATM phenotypes (38). The authors of this study additionally demonstrated that in obesity, myeloid specific *Irs2*^−/−^ mice exhibit impaired insulin sensitivity, associated with more pro-inflammatory (F4/80^+^CD11c^+^CD206^−^) and less anti-inflammatory (F4/80^+^CD11c^−^CD206^+^) ATMs. This suggests distinct differences between IRS2 and IR in regulating ATM phenotypes. These differences were explained by findings showing that IL-4 promotes M2 macrophage polarization through IRS-2 and post obesity, hyperinsulinemia through engagement of the IR, leads to macrophage IRS-2 downregulation (38). Further, several studies have identified myeloid dysfunctions associated with macrophage cell intrinsic insulin resistance. In this context, macrophages were rendered insulin resistant through pre-incubation with high-dose insulin, genetic deletion of the *INSR* or by pharmacologic inhibition of insulin signaling. Pre-treatment of macrophages with high-dose insulin leads to *INSR* downregulation and suppression of insulin signaling, which is also observed in freshly isolated macrophages from insulin-resistant mice, such as the leptin-deficient *ob/ob* mouse (49). In line, monocytes isolated from diabetic subjects show decreased surface expression and tyrosine kinase activity of the IR and diminished insulin-stimulated PI3K/AKT signaling (50). In response to free cholesterol (FC) loading, *Insr*^−^^/−^ macrophages exhibit attenuated AKT phosphorylation and an augmented ER stress response, that is independent of the degree of FC loading. This suggests macrophage PI3K signaling through the IR is required to withstand stressful stimuli. The functional consequences of this are increased apoptosis, unconnected to obvious changes in pro/anti-apoptotic gene expression. Indeed, western diet-fed mice with IR deficiency on an *Ldlr* deficient background in hematopoietic cells develop larger, more complex lesions with increased necrotic cores and apoptotic cells (40, 51). Furthermore, insulin resistant macrophages, post-transcriptionally upregulate CD36 and scavenger receptor A (SR-A), with increased CD36 protein levels dependent on defects in insulin stimulated PI3K signaling and proteasomal and lysosomal catabolism (40, 49). SR-A levels are coupled to increased ER stress as they are increased upon treatment with ER stress inducers (40). In agreement, primary *Insr*^−/−^ macrophages exhibit enhanced binding and uptake of modified LDL. Conversely, *in vivo* treatment of *ob/ob* mice with rosiglitazone, an insulin sensitizing agent and PPAR-γ activator, reverses this phenotype resulting in improved insulin signaling and decreased modified LDL uptake (49). Interestingly, in human macrophages, both CD36 and SR-A basal levels are reported to depend on PI3K activity as selective pharmacological inhibition of Class IA p110β or δ and Class IB p110γ attenuates their expression and is associated with reduced macropinocytosis and foam cell formation upon modified LDL challenge (52). Thus, although there may be species-specific differences, intrinsic murine myeloid cell insulin stimulated PI3K dependent signaling promotes myeloid cell survival and modulates lipid metabolism, decreasing foam cell formation. Consequently, cell intrinsic macrophage insulin resistance and associated downregulation of PI3K signaling results in elevated macrophage lipid burden and death, impacting ectopic lipid spillover, further contributing to pathogenesis in obesity.

## PI3Ks Promote Glucose Dependent Alternative Macrophage Activation

Hyperglycemia is a hallmark of T2D and glucose levels modulate intracellular macrophage metabolism through environmental glucose uptake and subsequent pyruvate and fatty acid generation and there are numerous excellent reviews on this topic (53, 54). Stable overexpression of GLUT1 in RAW264.7 macrophages promotes glucose uptake and metabolism (41). GLUT1, 3 and 5 expression increases as monocytes differentiate into macrophages and high expression is observed in foamy macrophages, which are typically found upon modified lipoprotein challenge and are reminiscent of ATMs (55–57). Enhanced glucose uptake might promote macrophage survival as *Insr*^−/^^−^ macrophages, exhibit increased cell death upon glucose deprivation (Figure 1) (40), an effect that could be particularly relevant in the context of modified lipoprotein presence (58). Interestingly, recent work indicates that GLUT1 and glucose transport is critical for the uptake of apoptotic cells (also known as efferocytosis), suggesting glycolysis may also promote anti-inflammatory phenotypes in macrophages in part through SLC16A1 mediated lactate release (Figure 1) (59). Other reports demonstrate that glucose promotes BMM proliferation and decreases LPS induced MHC-II expression, suggesting glucose levels might impact macrophage polarization (60). In line, high glucose levels have been described to induce the expression of Arg-1 and CD206 in macrophages in a PI3K dependent manner (61). Further, evidence of an importance for glucose in M2 responses is provided by studies demonstrating that PI3K-AKT dependent glucose utilization is critical for IL-4 responses (62, 63).

## PI3Ks can Influence FFA Signaling and ATM Accumulation

While physiologically FFA release through adipose lipolysis provides an important source of fuel, this process is dysregulated in the obese and insulin resistant state. The general dogma, particularly drawn from experimental murine studies, is that while unsaturated FFAs are anti-inflammatory, saturated FFAs are pro-inflammatory (64). Indeed, saturated FFAs such as palmitate promote skeletal muscle insulin resistance in part by blocking insulin mediated IRS-1 tyrosine phosphorylation and PI3K activity (65, 66). Given IRS-2 is predominantly expressed in macrophages (37), to our knowledge, no studies have addressed whether saturated FFAs decrease IRS-2 phosphorylation and render macrophages insulin resistant. Most studies utilizing macrophages in conjunction with palmitate have focused on its inflammation promoting effects. Indeed, palmitate triggered inflammation is JNK dependent, which is negatively regulated by PI3Ks (Figure 1) (67, 68). Although, palmitate has been suggested to mediate its effects via TLR4 (53), recent data indicates that JNK activation by palmitate is TLR4 independent (Figure 1). While LPS induced TLR4 signaling rapidly activates MAPK and NF-κB signaling and TLR4 endocytosis, palmitate activates these pathways much later and does not induce TLR4 endocytosis (54). The authors of this study demonstrated that LPS priming of macrophages altered cellular metabolism, gene expression and macrophage membrane lipid composition, which were necessary for palmitate induced inflammation (54). Notably, the effect of palmitate on inflammation might also depend on macrophage differentiation status. In fully differentiated macrophages, palmitate treatment elicits a pro-inflammatory phenotype, that is dependent on ER stress, as it is abrogated upon incubation with ER stress inhibitors (60). This is consistent with studies demonstrating that palmitate activates ER stress (64). However, during BMM differentiation chronic palmitate exposure been described to inhibit proliferation and promote an anti-inflammatory M2 phenotype, associated with increased PPAR-γ and CD206 expression (60).

Palmitate treatment of monocytes leads to macrophage inflammatory protein 1-alpha and beta upregulation (MIP-1α/β, also known as CCL3 and 4, respectively) and this occurs in a MAPK, NF-κB, and PI3K dependent manner indicating that PI3Ks can directly promote FFA mediated inflammation (69, 70). Interestingly, both chemokines are involved in neutrophil and monocyte recruitment, respectively (71), suggesting FFA mediated PI3K dependent signaling could promote increases in ATM number. Further evidence that palmitate mediated PI3K activation within myeloid cells regulates ATM content is provided by observations that palmitate treatment of macrophages induces netrin-1 and its receptor Unc5b, mediators that promote ATM retention and accumulation (72). Interestingly, in other cellular systems, netrin-1 acts in concert with its receptor in a PI3K dependent manner (73), although the functional relevance of PI3Ks to palmitate mediated ATM retention remains unexplored. These studies suggest that PI3Ks integrate signals derived from FFAs and thereby influences ATM accumulation and inflammatory status. However, many of the studies cited are limited by their exclusive use of *in vitro* models, disregarding the complexity of signals present *in vivo*.

## Cholesterol Activates the PI3K Pathway

Cholesterol exists as free cholesterol (FC) or as cholesterol esters. During obesity, adipose tissue accumulates FC and this correlates with increased ATM content (74). Cholesterol and modified lipoproteins are taken up by macrophages through macropinocytosis, scavenger receptors (e.g., CD36, SRA-1) and the low density lipoprotein receptor (LDLR), leading to foam cell formation that impacts inflammation and viability (Figure 1) (75). FC is reported to impact macrophage inflammation in a concentration dependent manner, with lower and higher levels promoting anti and pro-inflammatory phenotypes, respectively (76). In macrophages, FC also induces AKT phosphorylation indicating it activates the PI3K pathway (40). Macrophages use cholesterol efflux pathways to maintain cellular lipid homeostasis with ABCA1 mediating the transport of cholesterol and phospholipids to lipid-free apolipoproteins such as apoA-I (75). ABCA1 upregulation in turn selectively attenuates FC, dampening inflammation by reducing TLR trafficking to lipid rafts, indicating the presence of feedback loops that resolve inflammation (77).

## PI3K Dependent Uptake of Adipose Exosomes

Exosomes are small (30–150 nm) endosomal derived membrane microvesicles secreted from cells that carry proteins, lipids, nucleic acids, and can reprogram recipient cells (78). Recent work demonstrates that the uptake of adipose exosomes (AdExo), promotes BMM differentiation into ATM like cells by inducing lysosomal biogenesis (79). Interestingly, AdExo do not carry FFAs but are particularly rich in FC and triglycerides and are taken up by macrophages through macropinocytosis, PI3K dependently, suggesting PI3Ks might modulate macrophage lipid loading in response to AdExos (Figure 1). They thus represent a novel intercellular communication route for the transfer of these lipids to macrophages (79).

## PI3Ks Integrate the Environmental Cues That Dictate Macrophage Phenotypes in Obesity

Within adipose tissue during obesity, the metabolic stimuli outlined above, although elevated, likely exist at differing levels within the microenvironment and synergize their signaling with other stimuli, notably, LPS. This poses the question of how do myeloid cells respond to the combined actions of these stimuli and where do PI3Ks fit into this context during obesity? We propose a model where attenuated PI3K signaling within myeloid cells is central to meta-inflammation.

## How Might PI3Ks Affect the Synergy Between Metabolic Stimuli in Macrophages in Obesity?

**Figure 2:**
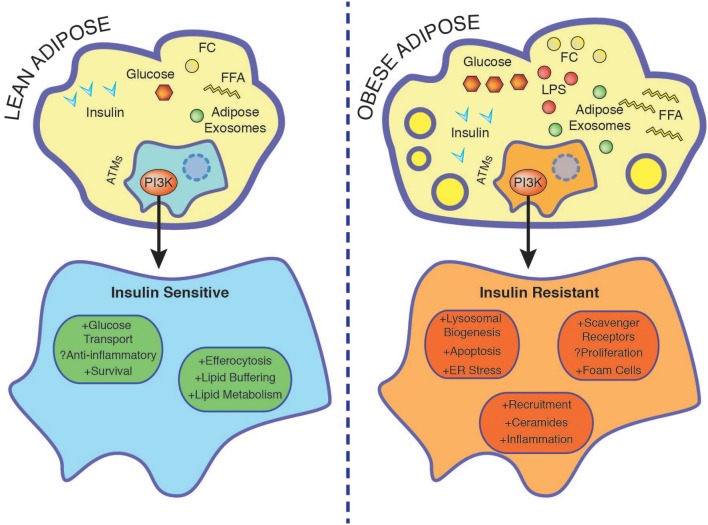
Nutrient accumulation during obesity is associated with rendering macrophage insulin resistant. In the lean state ATMs buffer lipids and insulin promotes survival through PI3K. Insulin resistant ATMs display enhanced foam cell formation associated with increased lysosomal biogenesis, ER stress, apoptosis, and exacerbated inflammation. See text for further details. ER, endoplasmic reticulum; FC, free cholesterol; FFA, free fatty acids; LPS, lipopolysaccharide.

Obesity alters the gut microbiome and is associated with increased circulating LPS, which initiates adipose tissue inflammation and macrophage activation in a manner dependent on intact TLR4 signaling, a phenomenon coined “metabolic endotoxemia” (80, 81). Interestingly, TLR4 ligation and palmitate presence synergistically augment macrophage ceramide production through *de novo* synthesis in the ER and this is implicated in augmenting IL-1β synthesis (82). This might be especially relevant given TLR4-dependent priming of macrophages is reported to be necessary for FFA induced inflammation and thus might act as a initiating stimulus promoting FFA mediated inflammation (Figure 2) (67, 83). Numerous studies demonstrate that ceramides alter PI3K signaling by promoting insulin resistance through either dephosphorylating AKT or through blocking AKT translocation to the plasma membrane (84–86). Together a potential synergy between LPS and FFAs might impact macrophage intrinsic insulin sensitivity through PI3Ks.

As TLR4 dependent signaling is increased in obesity, as outlined earlier, PI3Ks would presumably limit pro-inflammatory responses through various mechanisms including the promotion of M2 responses (13–16). Generation of alternatively activated macrophages would also be favored by prevalent hyperglycemia in obesity in a PI3K-AKT dependent manner (61–63). However, high doses of insulin render macrophages insulin resistant, decreasing PI3K signaling and thus inhibiting insulin stimulated glucose uptake through GLUT-1 (36). Together, decreased myeloid cell PI3K signaling in the insulin resistant state would shift macrophage phenotypes toward pro-inflammation through a synergistic effect of high insulin, glucose, and LPS.

High glucose/insulin/palmitate stimulation of macrophages leads to upregulation of lipid metabolism genes (ABCA1, CD36, and PLIN2) and cellular programs associated with lysosomal biogenesis and autophagy, mimicking the effects of FC (30, 75). FC activates the PI3K pathway and elevated AdExos in obesity are taken up by macrophages through macropinocytosis, PI3K dependently (40, 79). Decreased PI3K signaling in insulin resistant macrophages would therefore contribute to enhanced systemic levels of these metabolic stressors. Furthermore, attenuated PI3K signaling in insulin resistant macrophages leads to upregulation of scavenger receptors and compensatory proteasomal and lysosomal catabolism (40, 49). This in turn induces a vicious cycle of modified lipid uptake, further promoting ER stress and apoptosis, which is aggravated in insulin resistant macrophages (40, 58). Consequently, we propose in obesity, cell intrinsic macrophage PI3K signaling would be downregulated and result in elevated lipid burden and death. This would impact the lipid buffering capacity of ATMs, further promoting ectopic lipid spillover and meta-inflammation (Figure 2). To sum up, the different metabolic inputs outlined in this review affect the degree/strength of PI3K signaling and together synergistically determine macrophage cell survival, lipid metabolism, and inflammatory phenotype.

## Concluding Remarks

Slightly over 25 years ago, the concept of meta-inflammation was born by the discovery that adipose expressed TNF reduced adipose GLUT4 levels and neutralization of TNF in obese rats improved insulin sensitivity (5). Supportive of the key role of peripheral inflammation in obesity, obese myeloid-specific IKKβ or JNK deficient mice exhibit improved systemic insulin sensitivity (6, 7). Peripheral NF-κB activation is critical, as inhibiting this pathway in hepatocytes prevents IL-1β and insulin induced IR tyrosine phosphorylation and p85 association with IRS-1 (7). Since then, the contribution of ATMs to systemic inflammation has received much attention with the dogma that inflammatory pathways attenuate downstream PI3K signaling and initiate and exacerbate inflammatory responses, particularly in peripheral metabolic tissues such as the liver. However, only recently the importance of how environment re-programs and wires tissue resident macrophages has been appreciated (87).

We present an emerging paradigm where environmental stimuli encountered by ATMs during obesity reprogram them in a manner that is associated with macrophage intrinsic insulin resistance and drastic changes in intracellular lipids leading to oxidative and ER stress and upregulation of lysosomal and proteasomal programs. We propose myeloid cell PI3K activation integrates these environmental cues through its influences on saturated FFA responses, ATM accumulation, cell survival and the degree of lipid loading. This would presumably have consequences on ectopic lipid spill over and peripheral insulin sensitivity. While mice possessing global deletions of p85α/β and p55α/p50α exhibit improved insulin sensitivity (88–90) and mice with global deletions in p110α and p110β display impaired insulin sensitivity (91, 92), given that these subunits are deleted in all insulin sensitive tissues the exact function of myeloid cell specific PI3Ks during obesity and insulin resistance remains an enigma. To our knowledge, there is only one study that conditionally deleted a PI3K subunit in myeloid cells. By crossing floxed p110γ mice with mice expressing the Cre recombinase under the control of the *Tie*2 promoter, Breasson and colleagues demonstrated efficient deletion of p110γ in endothelial cells and adipose associated immune cells. These animals exhibited improved insulin sensitivity associated with increased CD206 expression in adipose tissue, independent of differences in ATM content, suggesting p110γ is dispensable for ATM recruitment but promotes M1 responses in obesity (93). Undoubtedly, myeloid cell specific deletions of class 1 PI3Ks in the context of obesity coupled to isolating primary macrophages from these mice and challenging them with the metabolic stimuli outlined in this review, will yield fruitful insights into the contribution of class I PI3Ks to obesity and ATM function.

## Author Contributions

OS, JB, AV, and GS has conceived and written this manuscript. JB designed the figures as presented.

### Conflict of Interest Statement

The authors declare that the research was conducted in the absence of any commercial or financial relationships that could be construed as a potential conflict of interest.
